# Uncommon *Candida* Species Fungemia among Cancer Patients, Houston, Texas, USA

**DOI:** 10.3201/eid2111.150404

**Published:** 2015-11

**Authors:** Dong Sik Jung, Dimitrios Farmakiotis, Ying Jiang, Jeffrey J. Tarrand, Dimitrios P. Kontoyiannis

**Affiliations:** Dong-A University College of Medicine, Busan, South Korea (D.S. Jung);; The University of Texas MD Anderson Cancer Center, Houston, Texas, USA (D.S. Jung, D. Farmakiotis, Y. Jiang, J.J. Tarrand, D.P. Kontoyiannis);; Brigham and Women’s Hospital/Dana-Farber Cancer Institute, Boston, Massachusetts, USA (D. Farmakiotis)

**Keywords:** Candida, cancer, candidemia, C. kefyr, C. lusitaniae, non-albicans, C. guilliermondii, C. lusitaniae, C. kefyr, C. famata, C. dublinensis, antimicrobial drugs, echinocandins, caspofungin, Houston, Texas, USA, fungi

## Abstract

Many uncommon *Candida* species that cause bloodstream infections (BSIs) are not well-characterized. We investigated the epidemiology, antifungal use, susceptibility patterns, and factors associated with all-cause death among cancer patients in whom uncommon *Candida* spp. BSIs were diagnosed at a cancer treatment center during January 1998–September 2013. Of 1,395 *Candida* bloodstream isolates, 79 from 68 patients were uncommon *Candida* spp. The incidence density of uncommon *Candida* spp. BSIs and their proportion to all candidemia episodes substantively increased during the study period, and the rise was associated with increasing use of echinocandin antifungal drugs. Thirty-seven patients had breakthrough infections during therapy or prophylaxis with various systemic antifungal drugs for >7 consecutive days; 21 were receiving an echinocandin. *C. kefyr* (82%), and *C. lusitaniae* (21%) isolates frequently showed caspofungin MICs above the epidemiologic cutoff values. These findings support the need for institutional surveillance for uncommon *Candida* spp. among cancer patients.

## Abstract 2

Potentially resistant bloodstream isolates and increasing use of echinocandins create a need for institutional surveillance for candidemia in cancer patients.

Despite the widespread use of antifungal prophylaxis and the introduction of new antifungal agents, the incidence of candidemia and associated mortality rates among patients with cancer remain relatively unchanged (*1*). In previous studies (*1*–*3*), >90% of all *Candida*-associated invasive fungal infections were caused by 1 of 5 *Candida* spp.: *C*. *albicans, C*. *glabrata, C*. *parapsilosis, C*. *tropicalis,* or *C*. *krusei*. However, the use of antifungal drugs such as azoles for prophylaxis and echinocandins that are being used more frequently among high-risk populations have been associated with a continuous shift from *C*. *albicans* to various non-*albicans Candida* spp. during the past 2 decades (*1*,*4*–*9*). Moreover, uncommon *Candida* spp. have emerged as causes of nosocomial bloodstream infections (BSIs) in studies of specific *Candida* spp. Those isolates commonly exhibit decreased in vitro susceptibility to antifungal agents (*10*–*15*).

The epidemiology and clinical features of many uncommon *Candida* spp. BSIs have not been well characterized. To that end, we evaluated the epidemiologic characteristics, susceptibility patterns, and factors associated with all-cause death among cancer patients who had uncommon *Candida* spp. BSIs. We also determined whether the increasing frequency of uncommon *Candida* spp. BSIs in the study cohort correlated with the increased use of specific antifungal agents.

## Patients and Methods

### Isolates

In this retrospective study, we examined the clinical microbiology database at the University of Texas MD Anderson Cancer Center (Houston, Texas, USA) to identify blood cultures that were positive for *Candida* spp. from patients ≥18 years of age during January 1998–September 2013. *Candida* isolates were grown on Sabouraud dextrose medium (37°C/48 h/200 rpm) and then phenotypically identified by using CHROMagar Candida medium (CHROMagar Company, Paris, France) and VITEK-2 YST (bioMérieux, Marcy l’Etoile, France). The identification methods were not changed during the study period. We excluded unidentified *Candida* spp. For our analyses, we selected only the first isolate recovered from blood if a patient had several blood cultures drawn that were positive for the same uncommon *Candida* spp. Antifungal susceptibility was tested by using the Clinical Laboratory Standards Institute broth microdilution reference method (*16*). The MIC for caspofungin was tested after March 2005 in the center. For uncommon *Candida* spp., other than for *C. guilliermondii*, clinical breakpoints are undefined; therefore, isolates that showed MICs higher than the epidemiologic cutoff value (ECV) were considered potentially resistant (*17*). There was no ECV for *C. famata*; therefore, those isolates were excluded from susceptibility comparisons.

### Data Collection

We retrospectively reviewed the electronic medical records of patients to obtain demographic, clinical, and laboratory data on the day of blood culture collection (Table 1); we also determined 28-day, all-cause mortality rates using a standardized electronic data collection form. Only first episodes of uncommon *Candida* spp. BSIs per patient were included in survival analyses. The study and a waiver of informed consent for anonymous data collection were approved by the Institutional Review Board of the MD Anderson Cancer Center.

**Table 1:** Characteristics of 68 cancer patients with candidemia caused by uncommon *Candida* species, Houston, Texas, USA*

Parameter	Result
Median age, y (range)	54 (19–82)
Male sex, no. (%)	39 (57)
Malignancy, no. (%)	
Leukemia	42 (62)
Lymphoma/multiple myeloma	9 (13)
Solid tumor	17 (25)
Charlson Comorbidity Index, median (range)	5 (2–10)
APACHE II score, median (range)	18 (3–39)
≥19, no. (%)	27 (40)
<19, no (%)	41 (60)
Intraabdominal source,† no. (%)	37 (54)
Central venous catheter, no. (%)	65 (96)
Corticosteroid-based treatment within 30 d before the day of blood culture collection, no. (%)	29 (43)
Chemotherapy within 30 d before the day of blood culture collection, no. (%)	51 (75)
HSCT, no. (%)	18 (27)
GVHD, no. (%)	10 (15)
TPN, no. (%)	12 (18)
Hemodialysis, no. (%)	10 (15)
ICU stay, no. (%)	35 (52)
Intubation, no. (%)	11 (16)
Neutropenia at onset, no. (%)	
ANC <500/μL	44 (65)
ANC <100/μL	40 (59)
Duration of neutropenia (<500/μL) before the day of blood culture collection, no. (%)	
1–14 d	22/44 (50)
15–28 d	8/44 (18)
>28 d	14/44 (32)

*Characteristics were recorded on the day of blood culture collection, unless otherwise specified. APACHE II, Acute Physiology and Chronic Health Evaluation II; HSCT, hematopoietic stem cell transplant; GVHD, graft-versus-host diseases; TPN, total parenteral nutrition; ICU, intensive care unit; ANC, absolute neutrophil count. †Intraabdominal source was defined as cases of abdominal surgery, gastrointestinal GVHD, peritonitis, cholecystitis, and cholangitis.

### Definitions

An episode of candidemia was defined as signs or symptoms of infection and >1 blood culture that was positive for *Candida* spp. Episodes were considered to be separate if they occurred ≥1 month apart. Breakthrough candidemia was defined as candidemia in a patient who had undergone therapy or prophylaxis with any systemic antifungal drug for >7 consecutive days before the index blood culture (*18*).

Neutropenia was defined as an absolute neutrophil count (ANC) of <500/μL, with further stratification at <100. Persistent neutropenia was defined as an ANC of <500 for >7 days. Neutrophil recovery was defined as restoration of the ANC to >500 for >3 consecutive days (*18*,*19*). The source of candidemia was considered to be intraabdominal if the patient had undergone abdominal surgery or had gastrointestinal graft-versus-host disease, peritonitis, cholecystitis, or cholangitis.

Catheter-related bloodstream infections were defined as described by Raad et al. (*20*) as 1) a colony count of blood obtained through the catheter hub that was >5-fold higher than that in blood obtained from a peripheral vein or 2) a catheter tip culture that was positive for *Candida* spp. The department of pharmacy provided defined daily doses according to the World Health Organization Anatomical Therapeutic Chemical classification system definition (http://www.whocc.no) for echinocandins, azoles, and amphotericin B (ampB) per 1,000 adult inpatient-days during the study period.

### Statistical Analysis

We used descriptive statistics to summarize the demographic, clinical, and outcome variables and the in vitro susceptibility data. We compared percentages with the χ^2^ test or Fisher exact test if the expected numbers were <5 in >20% of all cells. Poisson regression and the Cochran-Armitage test were used for the trend analysis of the annual BSI incidence densities and the proportions of candidemia caused by uncommon *Candida* spp., respectively. We also compared BSI incidence densities for 2 time periods—1998–2005 and 2006–2013—using Poisson distribution and test-based methods. The correlation between the annual use of antifungals and time was evaluated by using the Spearman correlation. The associations between the incidence densities of uncommon *Candida* spp. BSIs and the annual use of antifungals (defined as daily doses per 1,000 patient-days) were evaluated by using Poisson regression.

We used Cox regression analysis to identify factors that were significantly associated with death. Clinically relevant parameters in the univariate analyses (p<0.1) were included at model entry. The full model was reduced to a final model by using a stepwise elimination procedure. The proportional hazards assumption was tested graphically and by building time-dependent variables. Two-tailed p values <0.05 were considered statistically significant. All analyses were done by using SPSS statistical software version 21 (SPSS IBM, Armonk, NY, USA).

## Results

### Incidence Trends and Antifungal Use

We identified 1,395 blood cultures that were positive for *Candida* over the 16-year study period. We excluded 14 cultures that grew unspecified *Candida* spp. A total of 79 episodes of illness among 68 patients were caused by 5 uncommon *Candida* spp.: *C. guilliermondii* (n = 28, 41%), *C. lusitaniae* (n = 19, 28%), *C. kefyr* (n = 13, 19%), *C. famata* (n = 7, 10%), and *C. dublinensis* (n = 1, 1%). Patient demographic and clinical characteristics are shown in Table 1. Most patients had hematologic malignancies (n = 51, 75%). Of 44 patients who had low neutrophil counts, 40 were severely neutropenic (91%, ANC <100/μL).

**Figure 1:**
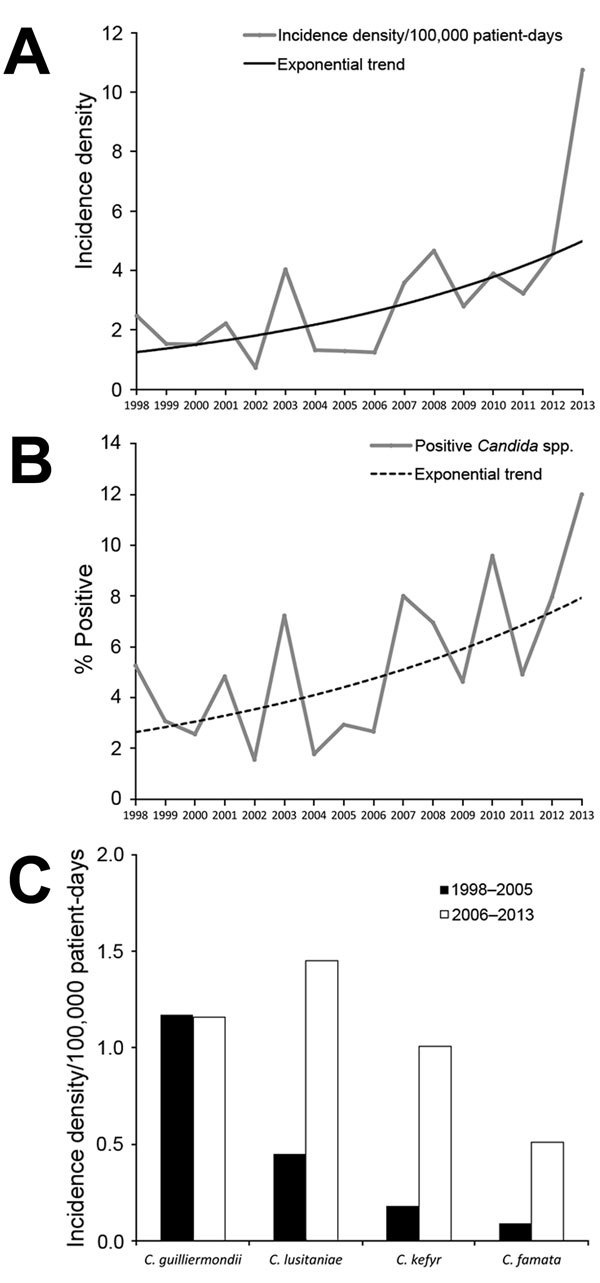
Increasing A) incidence density and B) proportion relative to all episodes of candidemia for bloodstream infections caused by uncommon *Candida* species at the University of Texas MD Anderson Cancer Center, Houston, Texas, USA, January 1998–September 2013. A) p<0.0001 and B) p = 0.001 for trend analyses. C) Incidence density of fungemia caused by uncommon *Candida* spp. during 1998–2005 compared with 2006–2013. There was a significant increase for *C. lusitaniae* (p<0.0001) and *C. kefyr* (p<0.0001) and a trend for increase for *C. famata* infections (p=0.068). *C. guilliermondii* infections remained stable.

The overall incidence of uncommon *Candida* spp. BSIs and their proportion relative to all episodes of candidemia increased significantly during 1998–2013 (incidence density p<0.0001; proportion p *=* 0.001) (Figure 1). The overall incidence density of uncommon *Candida* spp. BSIs was 3.17 episodes per 100,000 inpatient days, which increased from 1.89 (1998–2005) to 4.2 (2006–2013; p = 0.0001). The overall proportion of uncommon *Candida* spp. relative to all episodes of candidemia was 5.7% and increased from 3.6% (1998–2005) to 7.2% (2006–2013; p = 0.0004). During 2006–2013, *C. lusitaniae* had the highest incidence density (1.45 episodes/100,000 inpatient days), followed by *C. guilliermondii* (1.16), *C. kefyr* (1.01), and *C. famata* (0.51). The incidence density of candidemia caused by *C. lusitaniae* (p = 0.013) and *C. kefyr* (p = 0.01) increased significantly during 2006–2013 compared with that during 1998–2005; the incidence density of *C. guilliermondii* BSIs did not increase, and *C. famata* BSIs showed a trend for increase (p = 0.068) (Figure 1).

**Figure 2:**
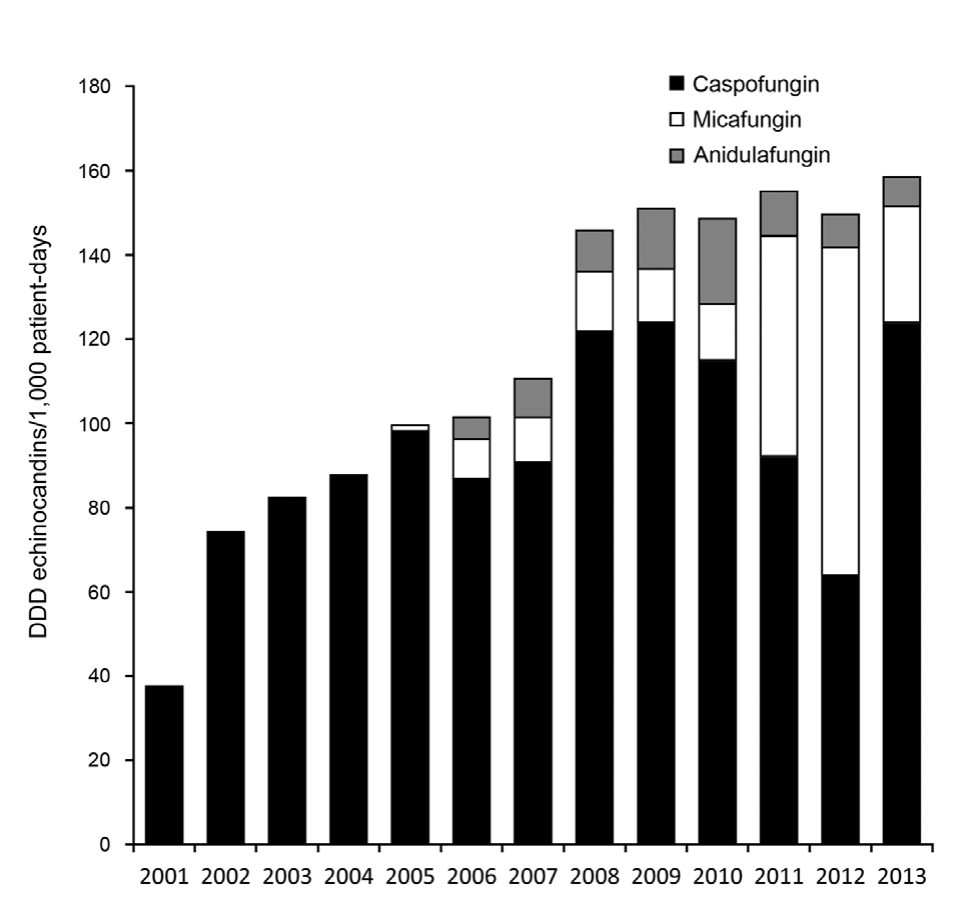
Increasing annual use of echinocandin antifungal drugs at the University of Texas MD Anderson Cancer Center, Houston, Texas, USA, January 2001–September 2013. Spearman’s correlation coefficient r = 0.98, p<0.0001. DDD, defined daily doses.

Echinocandins became available at the cancer center in 2001. The annual use of echinocandins increased significantly during 2001–2013 (Spearman r = 0.98; p<0.0001) (Figure 2), whereas annual azole and ampB use did not (data not shown). The increase in incidence density of uncommon *Candida* spp. BSIs was associated with the continuous increase in echinocandin use (p = 0.0062).

### Breakthrough Fungemia

Fungemia was detected in samples from 37 of 68 patients (54%) while they were being treated with antifungal agents, specifically with echinocandins (n = 21, 57%), ampB (n = 9, 24%), azoles (n = 6, 16%), or antifungal combinations (n = 1, 3%) (Table 2). Among 6 patients who experienced breakthrough fungemia during treatment with caspofungin, susceptibility data was available for 5 isolates; none were susceptible to caspofungin (MICs 4, 8, 8, 8, and 16 μg/mL). The most common species that caused breakthrough fungemia were *C. guilliermondii* (16/37 patients, 43%), *C. kefyr* (8/37 patients, 22%), *C. lusitaniae* (7/37 patients, 19%), and *C. famata* (6/37 patients, 16%). Most patients with breakthrough infections had underlying leukemia (33/37, 89%), compared with 9/31 patients (29%) who had no breakthrough infections (p<0.001), and neutropenia (31/37, 84%), compared with 13/31 (42%) who had no breakthrough infections (p<0.001). In addition, more patients who had breakthrough candidemia (26/37, 74%) than de novo candidemia (9/31, 29%) were admitted to the intensive care unit (ICU) (p = 0.001). The crude 28-day mortality rate among patients with breakthrough fungemia was 76% (28/37) (Table 2) and was significantly higher than that for patients with de novo candidemia (12/29, 41%; p = 0.005); Information regarding 28-day survival was available for 29 of 31 patients with de novo candidemia.

**Table 2:** Characteristics of 37 cancer patients with breakthrough candidemia caused by uncommon *Candida* species, Houston, Texas, USA

Parameter	Patients, no. (%)
Patients with leukemia	33 (89)
Patients with lymphoma or multiple myeloma	3 (8)
Breakthrough infection while receiving	
Amphotericin B	9 (24)
Echinocandin	21 (57)
Caspofungin	6 (29)
Micafungin	10 (48)
Anidulafungin	5 (24)
Azole	6 (16)
Fluconazole	2 (33)
Voriconazole	1 (17)
Itraconazole	3 (50)
Combination*	1 (3)
Species causing breakthrough fungemia	
* C. guilliermondii*	16 (43)
* C. kefyr*	8 (22)
* C. lusitaniae*	7 (19)
* C. famata*	6 (16)
Outcome of breakthrough fungemia	
Dissemination†	5 (14)
28-day crude mortality rate	28 (76)

*Caspofungin and liposomal amphotericin B. †Site of dissemination on autopsy: heart, lungs, and liver.

### In Vitro Susceptibility

In vitro susceptibility results were available for 57 isolates (Table 3). *C. guilliermondii* strains exhibited high rates of azole MICs above ECVs (fluconazole, 17%; voriconazole and posaconazole, 24%; Table 3). The 2 species that commonly were positive for caspofungin MICs above ECVs were *C. kefyr* (82% vs. 17% among other species; p<0.001) and *C. lusitaniae* (21%) (Table 3). Caspofungin MIC clinical breakpoints have been proposed only for *C. guilliermondii* (*17*); consequently, 13 *C. guilliermondii* isolates (87%) were susceptible to caspofungin (MIC <2 μg/mL), 1 was intermediate (MIC = 4 μg/mL), and 1 was resistant (MIC >8 μg/mL). One *C. famata* isolate had high caspofungin and fluconazole MICs (16 μg/mL for each). Even though ECVs for that species have not been defined, on the basis of ECV and clinical breakpoints for other *Candida* spp., that isolate could be considered azole/candin-nonsusceptible, making it multidrug resistant.

**Table 3:** Available susceptibility data for uncommon *Candida* isolates associated with fungemia among cancer patients, Houston, Texas, USA*

Medication	No. (%) cases; ECV, μg/mL				
	*C. guilliermondii*, n = 24 (41%)†	*C. lusitaniae*, n = 19 (28%)†	*C. kefyr*, n = 13 (19%)†	*C. famata*, n = 0 (10%)†	*C. dubliniensis*, n = 1 (1%)†
Amphotericin B					
No.‡	24	19	13	7	1
Wild type	24 (100); ≤2	19 (100); ≤2	NE	NE	0; ≤2
Non–wild type	0; >2	0; >2	NE	NE	1 (100); >2
Fluconazole					
No.	24	19	13	7	1
Wild type	20 (83); ≤8	16 (84); ≤2	12 (92); ≤1	NE	1 (100); ≤0.5
Non–wild type	4 (17); >8	3 (16); >2	1 (8); >1	NE	0; >0.5
Voriconazole					
No.	17	14	12	7	1
Wild type	13 (76); ≤0.25	13 (93); ≤0.03	10 (83); ≤0.015	NE	1 (100); ≤0.03
Non–wild type	4 (24); >0.25	1 (7); >0.03	2 (17); >0.015	NE	0; >0.03
Itraconazole					
No.	24	19	13	7	1
Wild type	21 (88); ≤1	19 (100); ≤0.5	NE	NE	1 (100); ≤0.25
Non–wild type	3 (12); >1	0; >0.5	NE	NE	0; >0.25
Posaconazole					
No.	17	14	12	7	1
Wild type	13 (76); ≤0.5	12 (86); ≤0.12	11 (92); ≤0.25	NE	1 (100); ≤0.12
Non–wild type	4 (24); >0.5	2 (14); >0.12	1 (8); >0.25	NE	0; >0.12
Caspofungin					
No.	15	14	11	7	1
Wild type	13 (87); ≤2	11 (79); ≤1	2 (18); ≤0.03	NE	1 (100); ≤0.12
Non–wild type	2 (13); >2‡	3 (21); >1	9 (82); >0.03	NE	0; >0.12

*Data were available for 57 of 68 isolates (24/28 *C. guilliermondii*, 0/7 *C. famata*). ECV, epidemiologic cutoff value (*17*); NE, not evaluable for susceptibility isolates because there are no defined ECVs for that species. †Numbers shown are number of isolates evaluable for susceptibility; percentages are percentage of isolates among all isolates.  ‡Results were the same by using a clinical breakpoint (MIC >1) or ECV.

### All-Cause Mortality

The all-cause 28-day mortality rate among this study cohort was 61% (40/66) (Table 4) and was positively associated with underlying leukemia, steroid exposure, ICU stay on the day candidemia was suspected and tested for, intubation, persistent neutropenia, high APACHE II scores (>19), hypoalbuminemia, and breakthrough fungemia (Table 5). We found no statistically significant association between all-cause deaths and specific *Candida* spp. or central venous catheter removal. In the multivariate Cox regression analysis, an ICU stay (adjusted hazard ratio [aHR] 4, 95% CI 1.8–9.05), persistent neutropenia (aHR 3, 95% CI 1.52–6.05), and a high APACHE II score (>19; aHR 2.8, 95% CI 1.39–5.78) were independently associated with the 28-day all-cause mortality rate (Table 5).

**Table 4:** Treatment and outcome of 68 cancer patients with candidemia caused by uncommon *Candida* species, Houston, Texas, USA*

Parameter	Value
Antifungal treatment	66 (97)
Amphotericin B–based regimen	5 (8)
Echinocandin-based regimen	13 (20)
Azole-based monotherapy	12 (18)
Combination antifungal treatment	36 (55)
Median duration of treatment, d (range)	16 (0–76)
Catheter-related infection	17 (25)
* C. guilliermondii*	6 (35)
* C. lusitaniae*	7 (41)
* C. famata*	2 (12)
* C. kefyr*	2 (12)
Central venous catheter removal, no. patients/no. in category (%)	41/65 (63)
Median time to central venous catheter removal, d (range)	3 (0–21)
Resolution of neutropenia, no. patients/no. in category (%)	15/44 (34)
Persistent neutropenia†	24 (35)
Growth factors	42 (62)
Leukocyte transfusion	5 (7)
Abscess drainage	3 (4)
Crude mortality rate at 28 d	40 (61)
Mortality rate by *Candida* spp.,‡ no. patients/no. in category (%)	
* C. guilliermondii*	16/27 (59)
* C. lusitaniae*	10/19 (53)
* C. kefyr*	10/13 (77)
* C. famata*	4/7 (57)

*Values are no. (%) patients except as indicated. †Persistent neutropenia was defined as an absolute neutrophil count of <500/mcL for ≥7days. ‡No available data for *C. dubliniensis*.

**Table 5:** Factors associated with 28-day crude mortality rate among cancer patients with candidemia caused by uncommon *Candida* species, Houston, Texas, USA*

Variable	Univariate analysis			Multivariate analysis	
	Hazard ratio (95% CI)	p value		Adjusted hazard ratio (95% CI)	p value
Underlying leukemia	7.6 (2.47–23.14)	<0.001		NR	
Steroid exposure	3.0 (1.03–8.71)	0.040		NR	
ICU admission	26.4 (6.42–108.55)	<0.001		4.0 (1.8–9.05)	0.001
Intubation	8.3 (1–69.64)	0.040		NR	
Total parenteral nutrition	4.0 (0.80–20.02	0.105		NR	
Persistent neutropenia†	30.6 (3.77–247.93)	<0.001		3.0 (1.52–6.05)	0.002
APACHE II score ≥19	12.8 (3.27–49.93)	<0.001		2.8 (1.39–5.78)	0.004
Hypoalbuminemia‡	3.5 (1.10–11.45)	0.030		NR	
Breakthrough fungemia	4.4 (1.53–12.64)	0.005		NR	

*NR, not retained in the multivariate analysis model; APACHE II, Acute Physiology and Chronic Health Evaluation II. †Persistent neutropenia was defined as an absolute neutrophil count of <500/μL for ≥7 days. ‡Serum albumin level <3.0 g/dL.

## Discussion

Comprehensive population-based registries of candidemia surveillance have documented the shift from human infections with *C. albicans* to non-*albicans* species over the past 2 decades (*4*,*21*,*22*). However, institutional surveillance is equally essential. For example, higher rates of echinocandin resistance are reported from oncology and transplantation centers in the United States (*23*–*25*) compared with population-based cohorts (*4*). At the MD Anderson Cancer Center hospital, the incidence of BSIs caused by uncommon *Candida* spp. and the proportion of those cases relative to all candidemia cases more than doubled during the past 16 years. Uncommon *Candida* spp. were frequently nonsusceptible to azoles and echinocandins and were commonly associated with breakthrough infections and high mortality rates. Notably, the incidence density for BSIs caused by uncommon *Candida* spp. was positively associated with the annual use of echinocandins.

Uncommon *Candida* spp. distributions vary by geographic region, patient population, and antifungal practices. In general, reported frequencies have been <10% among all *Candida* isolates (*21*,*22*,*26*,*27*), which is similar to the proportion of uncommon *Candida* spp. among all *Candida* BSIs (3.6%) during the first period of our study (1998–2005) and to that (3.3%) found in another study of cancer patients during 2009–2012 (*28*). Nevertheless, the proportion of uncommon *Candida* spp. BSIs relative to all episodes of candidemia in the MD Anderson Cancer Center hospital increased over the years, accounting for 12% of all *Candida* BSIs reported during 2013 (Figure 1), which is among the highest proportions reported to date. This striking difference reflects a severely immunocompromised patient population: 75% had hematologic malignancies, compared with 10.7% in the study by Tang et al (*28*). However, the most crucial determinant of this marked increase in uncommon *Candida* BSIs is likely the broad use of echinocandins. For example, in the study by Tang et al. (*28*), 88.8% of cancer patients with candidemia had previously received fluconazole and <2% had received an echinocandin; the opposite was true in our cohort, where almost one third of patients with uncommon *Candida* spp. fungemia had breakthrough infections while being treated with an echinocandin. Moreover, the incidence density of the uncommon *Candida* spp. BSIs in our study was positively associated with the increase in treatment with echinocandins.

In previous reports, *C. guilliermondii* was one of the most commonly isolated uncommon *Candida* spp. among patients with cancer (*9*,*13*,*29*); *C. dubliniensis* was common in the outpatient setting (*27*). Nevertheless, in our study, during the years 2006–2013, *C. guilliermondii* was not the most common isolate, and the incidence of *C. guilliermondii* fungemia did not increase substantially over the study period (Figure 2). This finding is in agreement with another study, wherein the increased use of echinocandins was not associated with an increase in the incidence of *C. guilliermondii* fungemia (*30*). The increase in the incidence of *C. kefyr*, predominantly among patients with hematologic malignancies, is in agreement with the results of another recent report (*12*), in which the increase was also attributed to the increasing use of the echinocandin drug micafungin. Taken together, those findings highlight the need, at an institutional level, to systematically monitor changes in *Candida* spp. distribution and the association with the selective pressure from antifungals.

The clinical features and outcomes of breakthrough candidemia with uncommon *Candida* spp. have not been well described. In our study, more than half of all patients with fungemia caused by uncommon *Candida* spp., and 36 of 51 patients who had hematologic malignancies (70%), had breakthrough infections. On the contrary, in a 1993–1998 candidemia study at our institution in which uncommon *Candida* spp. were excluded, ≈25% of all patients, and 46% of those with hematologic malignancies, had breakthrough infections (*31*). Nevertheless, the percentage of breakthrough infections among all *Candida* spp. BSIs (53%) in a more recent report (*32*) was almost identical to that in this study of fungemia caused by uncommon *Candida* spp. (54%). Those differences are further reflective of the changing epidemiologic characteristics of candidemia and the unique features of uncommon *Candida* spp. breakthrough infections, which seem to affect a more compromised patient population.

A direct comparison between common and uncommon *Candida* spp. was beyond the scope of this study, but in another report, among candidemic patients with acute leukemia, we observed a trend for higher mortality rates with the same uncommon *Candida* spp. infections on univariate analysis, but not on multivariate analysis (*25*). The only independent predictors of death in the study described here were ICU stay, persistent neutropenia, and high APACHE II score (Table 5), confirming that host characteristics are the most powerful predictors of response and should be adequately adjusted for in studies of candidemia outcomes.

We used the ECV to characterize uncommon *Candida* spp. bloodstream isolates as susceptible or potentially resistant, according to the updated Clinical and Laboratory Standards Institute/EUCAST definitions (*17*). *C. guilliermondii* strains exhibited high rates of azole resistance (Table 3), in agreement with the results of previous reports (*13*,*33*,*34*). However, echinocandin resistance among *C. guilliermondii* bloodstream isolates in our study was uncommon (a MIC >1 mg/L was observed for only 13% of isolates); in contrast, Girmenia et al. reported that a caspofungin MIC >1 mg/L was observed for 67% of *C*. *guilliermondii* strains (*13*). Moreover, the incidence of *C. guilliermondii* BSIs remained stable during the 16 years of our study (Figure 1) and was not substantially associated with echinocandin use. On the contrary, the most common species with caspofungin MICs above ECV was *C. kefyr* (82%); the incidence density for the species increased substantially over time (Figure 1) and was positively associated with the annual use of echinocandins (p = 0.004), but not azoles or ampB. Dufresne et al. (*12*) recently reported a similar rate (88%) of micafungin resistance (MIC >0.12 mg/L) in *C. kefyr* bloodstream isolates in patients with hematologic malignancies, possibly associated with institutional use of micafungin.

Our study has limitations that should be taken into consideration. First, it was a retrospective study from a single cancer center with a small number of episodes caused by individual uncommon *Candida* spp.; therefore, our observations might not be applicable to different patient groups at risk for uncommon *Candida* spp. BSIs. Second, uncommon *Candida* spp. were identified phenotypically, and it is possible that during the study period, some *C. dublinensis* isolates were identified as *C. albicans*, underestimating the frequency of that species. It should also be noted that with the introduction of molecular identification, the taxonomy of the *Candida* genus is in a state of change (*35*). The recent implementation of internal transcriber section sequencing (http://www.cbs.knaw.nl/databases, http://www.ncbi.nlm.nih.gov/genbank) and matrix-assisted laser desorption/ionization in mass spectrometry for *Candida* spp. identification are expected to further advance understanding of the epidemiology and clinical course of serious infections with uncommon *Candida* spp.

Third, we used in vitro caspofungin MIC alone to define echinocandin resistance, using no data on DNA mutations. However, there is evidence that caspofungin MIC interlaboratory variability may lead to incorrect categorization of susceptibility results (*36*), and micafungin and anidulafungin MICs correlate better with the presence of FKS mutations and clinical outcomes (*37*). Resistance to echinocandins emerges as a result of treatment and has been associated with mutations in FKS 1/2 genes, which encode the target enzyme for this specific class of antifungals, β-D-glucan synthase (*24*,*38*,*39*). In agreement with what we know about more common *Candida* spp., investigators have recently identified novel and established FKS1 gene mutations in *C. kefyr* clinical isolates that are associated with in vitro echinocandin resistance (*35*,*39*). Still, the spectrum of mutations that predispose patients to antifungal resistance, the role of epigenetic mechanisms, and the virulence of nonsusceptible, uncommon *Candida* strains (compared with wild-type) remain unknown at present. Therefore, some experts propose the concept of “clinical resistance,” which is a composite of factors related to the host, pathogen, and specific antifungal agent (*38*).

In summary, we observed a marked increase in the frequency of BSIs caused by uncommon *Candida* spp. in a contemporary series of patients with malignancies; those species were often associated with breakthrough infections and high mortality rates. The positive correlation between the increasing incidence of uncommon, potentially resistant *Candida* bloodstream isolates and the increasing use of echinocandins underscores the need for institutional surveillance and the rational use of antifungal drugs in cancer patients.
